# History of the Last Illness of Sir A. P. Cooper, Bart., and Examination of the Body after Death

**Published:** 1841-07

**Authors:** 


*History of the last illness of Sir A. P. Cooper, Bart., and examination of the body after death.*—For many months previous to his last illness, Sir Astley Cooper had occasionally experienced great dyspnœa, upon the slightest exertion; and it had been observed by his friends that the peculiarity of his complexion bespoke some serious impediment to the circulation. It was not, however, till about six weeks before his death that he found difficulty in assuming the recumbent posture; and about that time he began to pass the greater part of his nights in the arm-chair, rather than attempt to lie down. He still continued to see a few patients during the day, both at home and at their own houses. He now became the subject of frequent cough; which was immediately brought on, if he attempted to recline. The gout, of which he had for several years experienced periodical attacks, showed itself imperfectly in the fore-finger of the left hand; and his legs began to swell, owing to the depending position in which they constantly remained.

During all this time he refused medical aid; and it was not till the 22d of January that he consented to see any one, to whom he might state his symptoms. At the time he was first visited, he was sitting in his chair, with his body inclined forward, and his chin nearly resting on his chest; the pulse accelerated; not the slightest *bruit* nor abnormal sound in the heart, though the beat was extensive, and heard quite to the right side of the chest. The lungs afforded considerable bronchial rattle, but were neither consolidated nor compressed, and filled both cavities of the chest.

Although remedies appeared more than once to produce a temporary remission of his symptoms, and a further attack of gout in one foot seemed to afford some relief to the chest, yet, upon the whole, the disease advanced, and was attended by frightful fits of dyspnœa, during which his face was purple and his mind confused; and it was in one of these paroxysms that he died, on the morning of the 12th of February.

Shortly before his death, Sir Astley Cooper expressed a wish that the appearances which should be presented on the inspection of his body might be recorded in the Guy's Hospital Reports. He had particularly alluded to four points, the investigation of which he thought desirable;—a cured oblique inguinal hernia; a cured umbilical hernia; some suspected indications of phthisis in his youth; and an inability to sleep whilst lying on his left side.

*Examination of the body of Sir Astley P. Cooper, Bart., in the* 73*d year of his age, on February* 13*th*, 1841, *at* 9 *o'clock in the evening*, 32 *hours after death, by Mr. John Hilton, in the presence of Dr. Chambers, Dr. Bright, Mr. C. A. Key, and Mr. Edward Cock.*

The weather was warm and damp: there were slight cadaverous indications, from gravitation towards the posterior part of the corpse: the face and anterior part of the body exsanguine: there was a general and extensive œdema of the lower extremities; but no evidence of serous infiltration in the arms, nor in any other part of the surface of the body.

The head was not examined.

A globular projection, about the size of a large nut, was found at the umbilicus; which receded on pressure, leaving a well defined rounded aperture in the linea alba, capable of admitting the end of the little finger. This protrusion consisted of a few congregated lobes of fat placed immediately behind the umbilicus, between it and the peritoneum, the free surface of which was corrugated, and presented a puckered appearance, most probably inflammatory, and the result of the artificial curative means which had been employed for a long period during life.*

The anterior, thoracic, and abdominal parietes, were covered with a layer of fat, about an inch in thickness, soft, and oleaginous. The muscular tissue exposed during the inspection, was pale, soft, and flabby: indeed, the latter expression is applicable to nearly all the tissues. No gaseous or fluid effusion was found in the cavity of the peritoneum: the greater omentum, loaded with adipose matter, was contracted, and did not extend downwards more than two inches from the transverse colon. Some very old membranous adhesions existed between the right angle of the colon and the gall-bladder: cadaveric transudation of the bile from this viscus had slightly tinged the surrounding parts.

The viscera occupied their natural positions; excepting the cœcum, which was completely invested by the peritoneum, and hence less fixed than usual.

The liver healthy in form: some parts of its surface were slightly contracted and uneven; and sections of it presented hepatic venous congestion, approaching what is termed a "nutmeg appearance."

The gall-bladder was small; and contained a moderate quantity of healthy bile, which, upon gentle pressure, passed rapidly into the duodenum.

The spleen was rather larger than natural, its capsule a little opaque, and the interior of the organ very firm; a section presenting a smooth solid surface of a purplish gray colour.

The stomach was large and distended with gas; the cardiac extremity stained brown by cadaveric transudation, or the action of the gastric fluid upon the blood; its tissues appeard quite healthy.

The small intestines presented nothing abnormal: nor was there any thing remarkable in the large intestines, excepting the dilated condition of the cœcum, the parietes of which were thin; its mucous membrane congested.

The pancreas was healthy.

The kidneys were surrounded by a considerable quantity of adipose tissue, remarkably dense, and very firmly adherent to the fibrous capsule of the gland. Both kidneys were much congested with blood, rather larger than natural, their surfaces mottled, and slightly granular. These morbid conditions

---

*Sir Astley Cooper wore a piece of cork adapted to the umbilicus; and maintained in its place by straps of adhesive plaster, during many years, and until his fatal illness.

were the most evident at the lower part of the left kidney; less advanced but more generally diffused, in the right: and on the anterior surface of the latter, near its convex edge, were found too small cysts, containing a straw-colored fluid.

The supra-renal capsules were healthy.

The urinary bladder was healthy and contracted, and contained about two drachms of whitish turbid urine.

The internal abdominal ring, on the left side, was rendered distinct by a tubular extension of the peritoneum for about an inch into the inguinal canal.

A depression existed in a corresponding situation on the right side, the bottom of which was firm, irregular, and corrugated; and upon very careful examination, a minute serous canal, not more than a line in breadth when opened, was traced extending from it, along the spermatic cord, into the cavity of the tunica vaginalis, being the remains of a congenital inguinal hernia.*

Upon raising the sternum and cartilages of the ribs, both lungs were brought into view; and retained their expanded condition, overlapping the pericardium, and manifesting no disposition to collapse. No pleuritic adhesions existed on either side of the chest; nor was there any effusion, except into the right pleural cavity, which contained about three ounces of sanguinolent rather turbid serum.

A little recent pleuritis was found on the middle lobe of the right lung, rendering it sligntly adherent by plastic effusion to the adjoining ribs to a small extent. Both lungs presented general vesicular emphysema to a very great degree, and their edges were more rounded than natural.

The larynx was not examined.

The lining membrane of the trachea and larger bronchi was smooth, but of a dark purple hue, from congestion in the minute bloodvessels: the same appearances extended throughout the bronchial ramifications, the smaller of which were filled with a very tenacious puriform mucus; and many of them were observed much dilated. Both lungs were extremely congested with dark blood, especially in and near the central portions of their lobes. At the superior and posterior part of the right lung was a small depressed and somewhat contracted surface, about the extent of a sixpence; a section of which exposed a calcerous mass, very uneven upon its surface, and about equal to the size of a small pea: it was placed about three lines distant from the pleura.

---

*Sir Astley Cooper wore a truss on the right inguinal canal, from the age of nineteen to twenty-five.

When the pericardium was opened, the heart was seen, very large and distended; and about two ounces of rather dark or brown-colored slightly turbid serum occupied the posterior part of the cavity.

The right auricle and ventricle filled with very dark-colored imperfectly-coagulated blood. The auriculo-ventricular valves sound. Through one of the pulmonary valves, near its angle of union with an adjoining valve, was a perforation nearly the size of a small goose quill. A tolerably firm fibrinous coagulum was found in the pulmonary artery and its branches, extending, by minute prolongations, to the fifth divisions; these were made evident, by withdrawing them in a continuous mass with the forceps.

The left auricle and ventricle were occupied by a large quantity of black grumous half-liquid blood. A large portion of the mitral valve opaque, and a little thickened; otherwise healthy. The aortic valves thickened, and rather rigid at their attached margins; whilst the free margins presented a remarkably healthy appearance for their age.

The left ventricle was much dilated; its apex much broader, and more prolonged than natural: the parietes somewhat hypertrophied; and the muscular fibres of the whole organ were pale, flabby, and weak.

The aorta, which was small and narrow, pursued its usual course, but gave off the left vertebral artery between the left common carotid and left subclavian. The entrance to the arteria innominata was contracted, and slightly irregular.

Many small irregular yellowish opaque patches were seen under the lining membrane of the thoracic aorta and the ascending portion of the left subclavian artery. In the midst of the parts so affected, the internal membrane was much softened, breaking down under slight pressure: at three or four points it was destroyed to a small extent, admitting a thin layer of dark matter, probably altered blood, separating it in a slight degree from the subjacent tissue: this latter state was noticed near the origin of the arteria innominata and the commencement of the descending aorta. The whole length of the abdominal aorta was full of black grumous blood; its parietes thickened; the lining membrane opaque, and raised by the sub-deposition of hard, almost bony matter.—*From Guy's Hospital Reports, in London Med. Gazette.—Med. Exiner.*

*Principal circumstances connected with seven cases, in which an Artificial Anus has been established in the adult for the relief of urgent symptoms produced by Stricture of the Rectum.* By W. H. Walshe, M. D.

[1. Fine (An. ?) *Vid Odier, Man. de Medecine*, p. 274, ed. 2, 1818.]—Female, æt. 70. Symptoms of gangrene from fæcal retention; tumour completely obliterating the rectum at its origin.

Opening made at "most prominent part of the abdomen;" one or two stools daily afterwards; patient lived more than a year, and died dropsical.

[2. Pillore (1776.) *Vid. L'Experience, Janiver* 30, 1840.]—Adult male. No stool for upwards of a month; not a particle of two pounds of mercury, which had been exhibited by the mouth, discharged. Eight or nine inches of the end of the colon and beginning of the rectum totally obstructed from schirrhous induration, &c.

Transverse incision of the integuments above the fold of the right groin, and same of the cæcum; abundant fæcal evacuation. Peritonitic symptoms on the 20th; death on the 28th day. The bowels had not been completely emptied; the exact original weight of mercury being found in a knuckle of the jejunum, behind the bladder.

[3. Freer (1817.) *Vid. Med. and Phys. Journal*, vol. 45, p. 9, 1821.]—Male, æt. 47. Contraction of gut complete; attempt made to divide the stricture; no stool for ten days, vomiting, hiccup, &c.; "death inevitable."

Incision above and within anterior superior spine of the left ilium, and in descending colon; painful evacuation; patient, however, gradually sank, and died on the ninth day.

[4. Pring (1820.) *Vid. Med. and Phys. Journal*, vol. 45, p. 1, 1821.]—Widow, æt. 64. Obstruction seven inches above the anus; total retention of fæces for twelve days.

Incision within and above antero-superior spine of the left ilium, and in descending colon; immediate and forcible discharge of fæces. Three months after, indurated fæces passed through the natural anus, and continued to do so. Six months after, the patient was in good health.

[5. Martland (1824.) *Edinb. Med. and Surg. Journ.* p. 271, 1825.]—Male, æt. 44. A large tumor protruding, as it were, from the neck of the bladder; no stool for twenty-six days; marked stercoral tympanites.

Incision an inch above and within antero-superior spine of the left ilium, and in descending colon; instant escape of fæces and flatus; a year after the patient enjoyed good health; soft stools passed on a few occasions at one period through the natural anus.

[6. Amussat (1839.) *Vid. Gazette Medicale, Oct.* 1839.]—Woman, æt. 48. No stool for upwards of *twenty-six* days; nausea, vomiting &c. Hard, round, immoveable tumours, at the upper part of the rectum.

Incision in left lumbar region, and in descending colon. Four months after the operation, patient in excellent health; one or two evacuations daily; flatus passed by natural anus.

[7. Amussat (1839.) *Vid.* same paper.]—Case previously treated by breaking down fungous masses in the rectum, and subsequently by cauterization. Patient in a deplorable state.

Opening made in left lumbar region and in descending colon; no escape of fæces till the fifth day. Four months after, the patient in a better state than before the operation, and about to quit Paris for the country.

"When the disease is cancerous, the chances of ultimate advantage are, of course, vastly less than in cases of retention from simple induration; but even here it may be justifiably performed, provided the patient, after having been made fully acquainted with the nature and likelihood of the benefit to follow, still desires to undergo it." It is scarcely necessary to add, in defence of this position, that (even admitting the benefit obtained to be necessarily of short duration, which is by no means certain,) there are cases wherein the preservation of a given life, even for a few days, may be of the utmost consequence to families, in a worldly point of view.

It will be observed that in Pillore's case the unfortunate issue probably depended on the obstruction caused by the gravitation of the mercury in the pelvis: the site of the incision was also evidently ill-judged. The cause of death in Freer's case does not very clearly appear from his details.

M. Amussat's motives for preferring the operation proposed by Callisen (the lumbar incision, whereby implication of the peritoneum is avoided) to the inguino-abdominal incision of Littre, are explained in his papers referred to.—*London Med. Gaz.—Ibid.*

